# Embryonal tumor with multilayered rosettes: Post‐treatment maturation and implications for future therapy

**DOI:** 10.1002/cnr2.1812

**Published:** 2023-03-25

**Authors:** Francesca M. Gualano, Patrice Hassoun, Claire L. Carter, Derek Hanson

**Affiliations:** ^1^ Department of Pathology Hackensack Meridian School of Medicine Nutley New Jersey USA; ^2^ Hackensack Meridian Center for Discovery and Innovation Nutley New Jersey USA; ^3^ Department of Pathology Hackensack University Medical Center Hackensack New Jersey USA; ^4^ Department of Pediatrics, Joseph M. Sanzari Children's Hospital Hackensack University Medical Center Hackensack New Jersey USA; ^5^ Department of Pediatrics Hackensack Meridian School of Medicine Nutley New Jersey USA

**Keywords:** embryonal tumor with multilayered rosettes, ETMR, post‐treatment differentiation, post‐treatment maturation

## Abstract

**Background:**

Embryonal tumor with multilayered rosettes (ETMR) is a deadly grade IV pediatric brain tumor. Despite an intensive multimodal treatment approach that includes surgical resection, high‐dose chemotherapy, and radiotherapy, the progression‐free survival at 5 years is less than 30%.

**Case:**

We report a case of long‐term survival in a 5‐month old female with a large mass in the posterior fossa, diagnosed as ETMR, which subsequently underwent treatment‐induced maturation. Prior to chemotherapy, histopathology revealed an abundance of highly proliferative, undifferentiated cells and multilayered rosette structures. Conversely, post‐treatment histopathology revealed cell populations that differentiated into neuronal and ganglionic phenotypes. At 5‐year follow‐up, the patient remains progression‐free.

**Conclusion:**

This finding contributes to the few reports to date of post‐treatment differentiation/maturation of ETMR cell populations, with an implication for less cytotoxic therapeutic interventions aimed at differentiation.

## 1 INTRODUCTION

Embryonal tumor with multilayered rosettes (ETMR) is a deadly pediatric brain tumor that predominantly affects infants younger than 3 years old and has a 5‐year survival rate between 0% and 30%.^1^, ^2^ ETMR has low intertumoral genetic heterogeneity, is characterized by a hallmark amplification of the C19MC miRNA cluster on Chromosome 19q13.42, and has high expression of the RNA‐binding protein, LIN28A.^3^, ^4^ The histological signature of ETMR is multilayered rosettes, which are remnants of the undifferentiated neural tube, suggesting a prenatal oncogenic transformation that results in embryonal progenitor and progenitor‐like cells.^5^

Stalled developmental programs were recently described as the root cause of pediatric brain tumors, including ETMR. Jessa et al.^6^ mapped bulk and single‐cell transcriptomes of ETMR and other pediatric brain tumors, juxtaposing this data to reference datasets taken from human fetal brain and their developmental atlas derived from the analysis of cell types and differentiation pathways in the prenatal mouse and human brainstem. While the cell of origin for ETMR was postulated to derive from an oncogenic transformation in prenatal radial glia cells, the bulk tumor microenvironment mapped to a spectrum of neuronal lineage populations, including a progenitor‐like phenotype, a migratory phenotype, and a more mature phenotype.^6^ The progenitor‐like phenotype expressed the *TTYH1*‐C19MC fusion, whereas this promotor region was found to be silenced in malignant cells that expressed neuronal differentiation markers.^6^ The authors of this study provided a foundational framework for targeting restricted developmental pathways to overcome the impaired differentiation observed within ETMR and other pediatric brain tumors.^6^

Herein, we report a case of long‐term survival (greater than 5 years) in a patient with ETMR, whose post‐treatment histopathology revealed maturation of undifferentiated embryonal cells into mature neuronal and ganglionic phenotypes. This case is important, because it builds upon the postulation made by Jessa et al. and others supporting differentiation as a mechanism for treating malignant, embryonal tumor cell populations, demonstrating the feasibility of treatment‐induced differentiation for prolonging patient survival. Furthermore, we review the literature on post‐treatment differentiation of ETMR, of which there are only three other publications, as well as other pediatric brain tumors. These reports on treatment‐induced differentiation support the notion of differentiation as a promising therapeutic aim for novel drug development for the treatment of deadly pediatric brain tumors.

## 2 CASE REPORT

**FIGURE 1:**
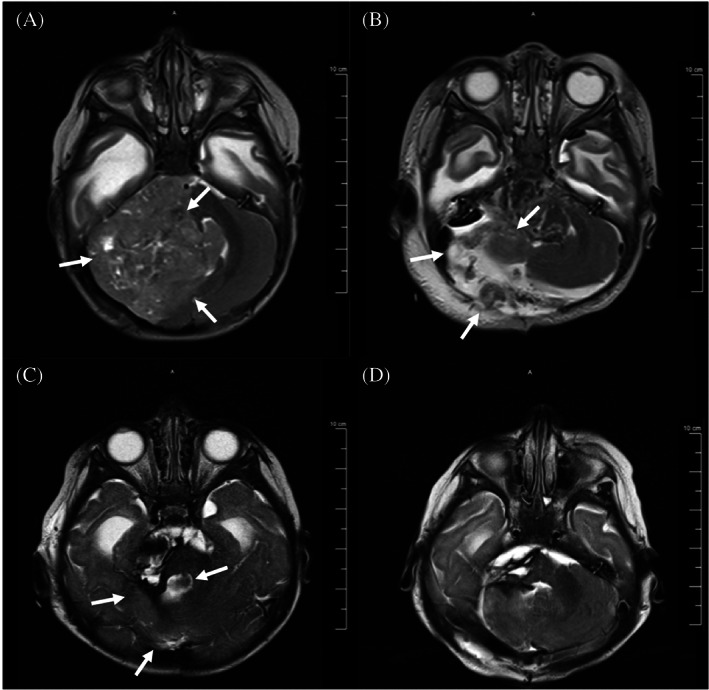
Axial T2 MRI, here depicting the level of the posterior fossa: (A) tumor at diagnosis, 6.5 × 6.5 × 4.4 cm (margins portrayed by white arrowheads) (December 2016); (B) residual tumor following the initial surgical resection, 4.9 × 4.2 × 4.3 cm (margins indicated by white arrowheads) (December 2016); (C) residual tumor following the fourth cycle of chemotherapy, 4.5 × 2.4 × 2.3 cm (margins indicated by white arrowheads) (May 2017); and (D) following gross total resection of residual tumor (June 2017).

**FIGURE 2:**
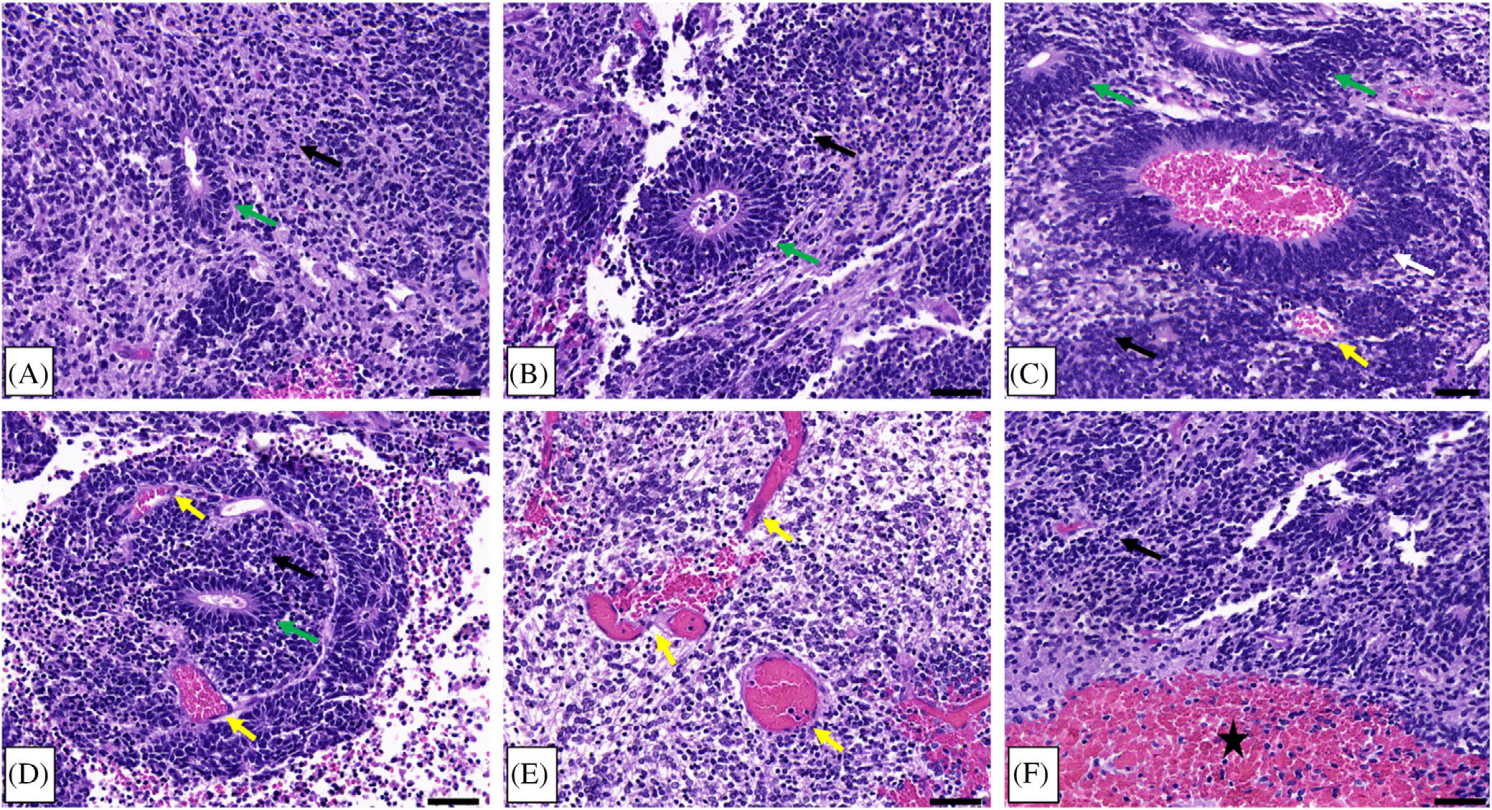
Representative histopathology characteristic of ETMR. Hypercellular small blue embryonal cells with scant cytoplasm are shown throughout the tumor (black arrows, A–F). Multilayered rosettes with a clear central lumen or a central lumen with cellular debris (green arrows, A–D). A large perivascular pseudorosette is shown (white arrow, C), along with multiple vascular formations (yellow arrows, C–E), and extensive hemorrhage (black star, F). Scale bars = 50 μm.

In 2016, a 5‐month‐old female presented to the Emergency Department of the Joseph M. Sanzari Children's Hospital at Hackensack University Medical Center with vomiting and somnolence. After ruling out infectious and metabolic differentials with diagnostic laboratory tests, an MRI of the brain was obtained and showed a 6.5 × 6.5 × 4.4 cm calcified, heterogeneously enhancing mass in the right posterior fossa (Figure 1A). Subsequent to subtotal resection, the residual tumor size was 4.9 × 4.2 × 4.3 cm (Figure 1B). Histopathology confirmed the presence of hypercellular areas of embryonal cells with minimal cytoplasm; abundant multilayered rosettes, vascular proliferations, and hemorrhage were detected throughout the tumor (Figure 2). Perivascular pseudorosettes were also noted. Immunohistochemistry (IHC) showed that tumor cells were vimentin, synaptophysin, and INI‐1 positive and GFAP negative. These results were consistent with ETMR. At the time of the case, the *2016 WHO Classification of Tumors of the Central Nervous System* had just defined ETMR as a single entity, and diagnosis required detection of the amplification of the C19MC miRNA cluster on Chr19q13.42. IHC staining for LIN28 was also characteristic of an ETMR diagnosis. Patient samples were sent to NYU Langone Health (New York, NY) and the Hospital for Sick Children (Toronto, Ontario, Canada) for confirmatory diagnostics. Methylation profiling and positive LIN28 IHC staining confirmed the diagnosis of ETMR.

Following diagnosis, the patient underwent 4 cycles of induction chemotherapy, according to the DFCI‐IRS‐III regimen.^7^ Following the fourth chemotherapy cycle, MRI showed a 72% decrease in the tumor size (Figure 1C), and a gross total resection of the tumor was performed thereafter (Figure 1D).

### 2.1 DFCI‐IRS‐III chemotherapy induction regimen

The chemotherapy induction regimen based upon the DFCI‐IRS‐III approach, as detailed by Hanson et al.^7^ included the following drugs, drug doses, and routes of administration: Vincristine 2 mg/m^2^ i.v.; Cisplatin 90 mg/m^2^ i.v.; Doxorubicin 30 mg/m^2^/day with continuous infusion over the course of 48 h; Cyclophosphamide 300 mg/m^2^/day with continuous infusion over the course of 72 h and Cyclophosphamide 600 mg/m^2^ i.v.; Etoposide 100 mg/m^2^ i.v.; Actinomycin D 1.2 mg/m^2^ i.v.; and Topotecan 0.2 mg/m^2^ intrathecally. Regarding the timepoints of administration, Vincristine was administered on day 1 of weeks 1–13 and week 16 of therapy.^7^ Cisplatin was administered on day 1 of weeks 1, 4, 7, and 10.^7^ Doxorubicin was administered on days 2 and 3 of weeks 1, 4, and 13.^7^ Cyclophosphamide at 300 mg/m^2^/day was administered on days 2, 3, and 4 of weeks 1, 13, and 16.^7^ Cyclophosphamide at 600 mg/m^2^ was administered on day 2 of weeks 7 and 10.^7^ Etoposide was administered on days 1, 2, and 3 of weeks 4, 7, and 10.^7^ Actinomycin D was administered on days 1, 2, 3, 4, and 5 of week 16.^7^ Topotecan was administered on day 1 of weeks 1, 2, 4, 7, and 13.^7^

### 2.2 Defibrotide treatment

An adverse effect of this patient's chemotherapeutic course was sinusoidal obstructive syndrome, which was rapidly responsive to treatment with defibrotide. Defibrotide is a polydisperse polydeoxyribonucleotide that has thrombolytic effects that are beneficial for the hypercoagulable sequelae of cancer states, and it also demonstrated anti‐tumor activity when used as a single agent against multiple myleoma.^8^ Recently, Dong et al.^9^ has shown defibrotide to modulate brain metastasis through inhibition of “proliferation, migration, invasion, and promotion of lactate dehydrogenase release of brain metastatic tumor cells, and elevation of the levels of blood brain barrier tight junction proteins and metastasis‐related proteins” via activation of adenosine A2A receptors and downstream inhibition of the SDF‐1/CXCR4 axis. Lastly, defibrotide was shown to have anti‐angiogenic properties after inhibiting the formation of new blood vessels during in‐vitro and in‐vivo studies and was postulated for use as an adjuvant anti‐cancer therapeutic.^10^ We are uncertain how this drug impacted the disease course in the patient presented here; however, defibrotide is noteworthy to consider for further investigation in embryonal CNS tumors.

### 2.3 Pre‐ and post‐treatment pathology demonstrating maturation

**FIGURE 3:**
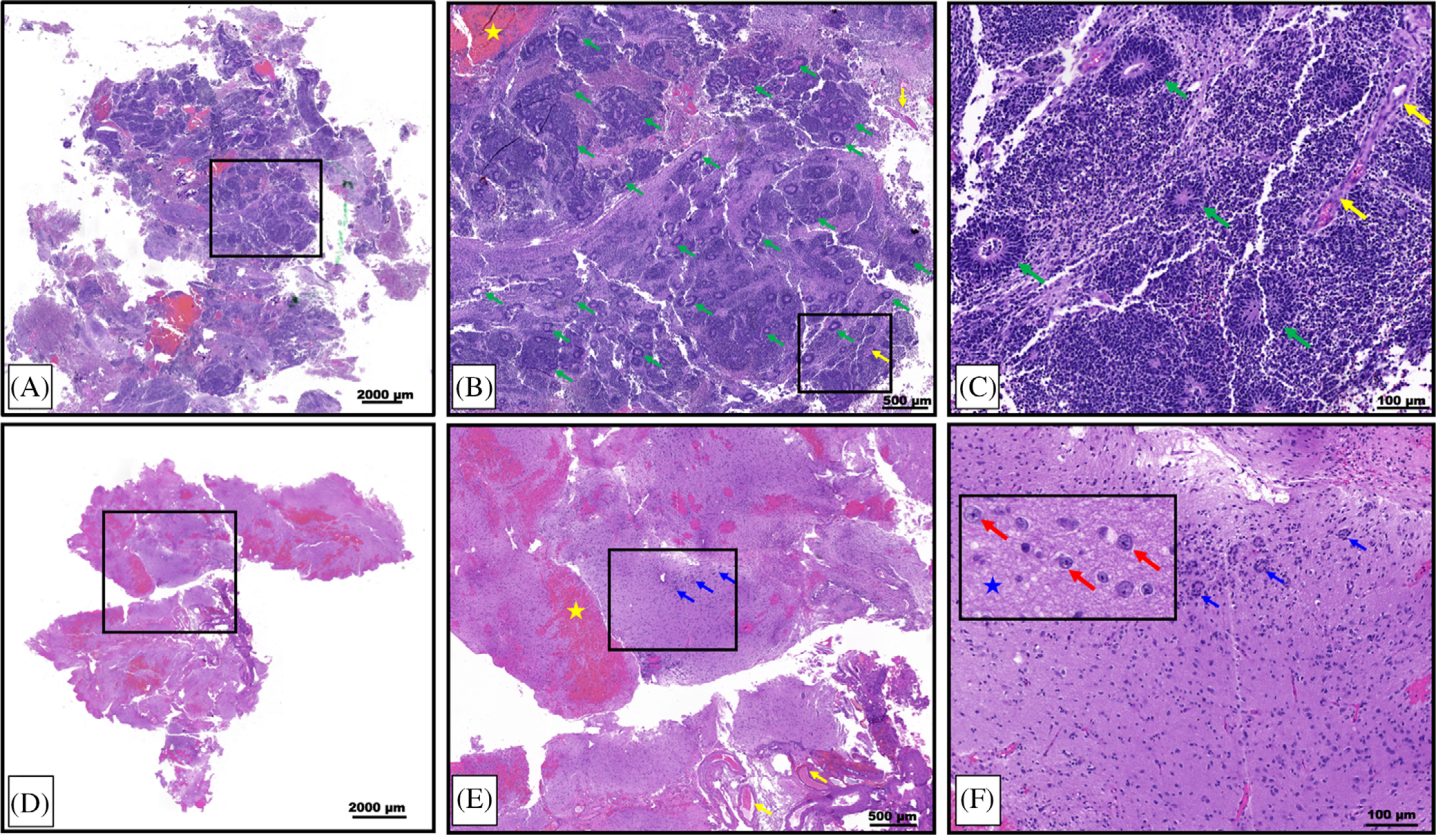
Pre‐treatment: (A) overview of a tumor tissue specimen from the pre‐chemotherapy resection, showing high cellular density and abundant multilayered rosettes, with areas of hemorrhage. (B) Higher magnification region taken from the black box shown in (A), showing a plethora of primitive, multilayered rosettes (examples shown with green arrows) within regions of hypercellular embryonal small blue cells with scant cytoplasm, as well as angiogenesis and hemorrhage (yellow arrows and star, respectively). (C) Higher magnification region taken from the black box shown in (B). Characteristic multilayered rosettes of ETMR (green arrows) in a dense background of undifferentiated, small, round, blue cells and areas of angiogenesis (yellow arrows). Post‐treatment: (D) overview of a post‐chemotherapy resection of residual tumor tissue, showing an observable, lower cellular density and an abundance of neuropil. (E) Higher magnification region taken from the black box shown in (D). Small monolayered rosettes (blue arrows) in a background of neuropil, lower cellular density, less mitotic activity, less atypia, and differentiated cells; regions of angiogenesis and hemorrhage are present (yellow arrows and star, respectively). (F) Higher magnification region taken from the black box shown in (E). An abundance of differentiated neuronal cells (inset, red arrows) that were absent in the pre‐treatment pathology, with abundant neuropil (inset, blue stars). Small monolayered rosettes are present (blue arrows).

**FIGURE 4:**
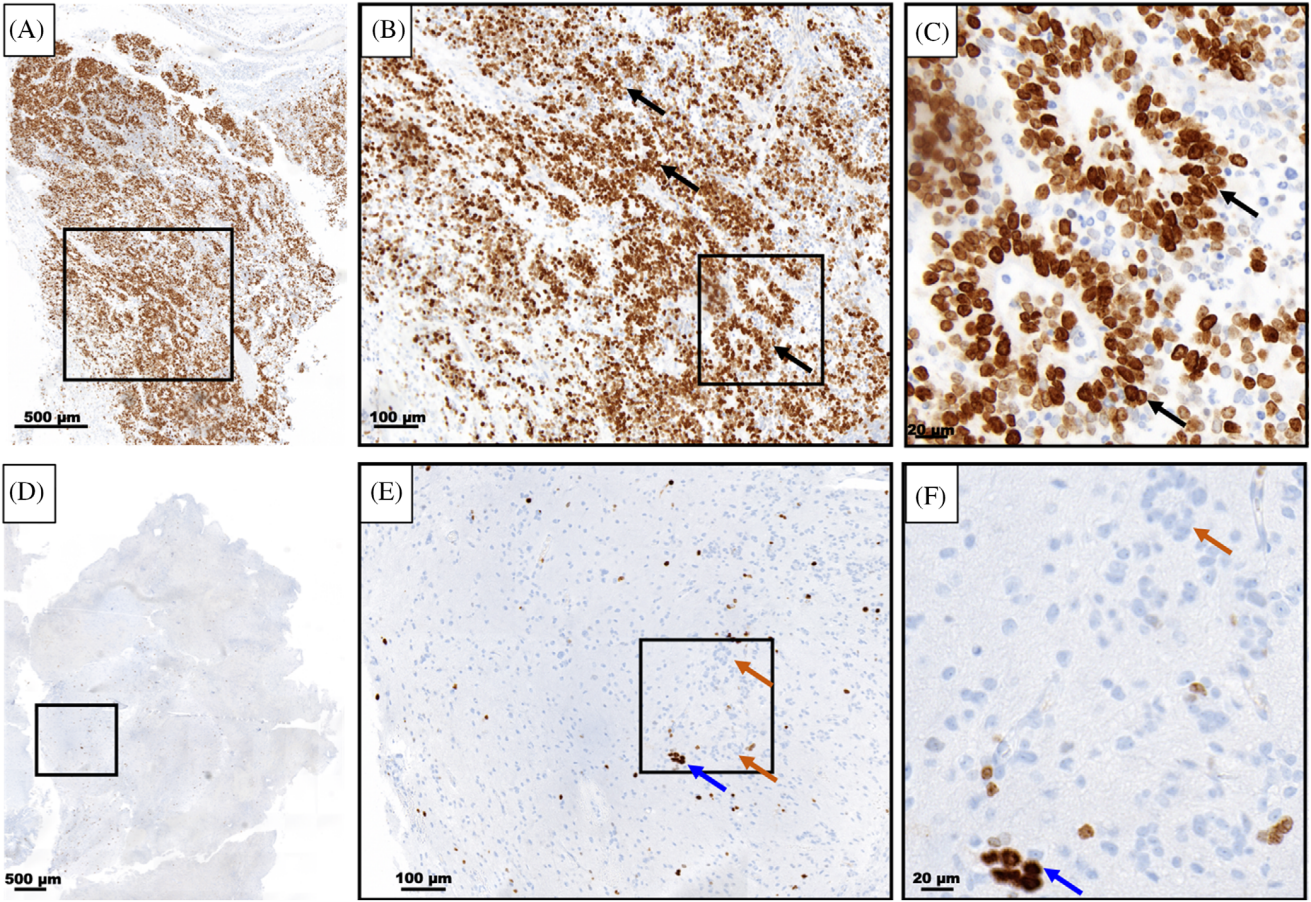
Pre‐treatment: (A) overview of Ki‐67‐stained tissue from pre‐chemotherapy tumor specimen, with an observable high‐density of positive immunoreactivity. (B) Higher magnification region taken from the black box shown in (A). High Ki‐67^+^ proliferation index throughout and in the cells of the multilayered rosettes (black arrows). (C) Higher magnification region taken from the black box shown in (B) demonstrates Ki‐67^+^ cells in the multilayered rosettes and in the cellular contents of the rosette lumen. Post‐treatment: (D) overview of Ki‐67‐stained tissue from a post‐chemotherapy residual tumor specimen, with a noticeably lower Ki‐67 positive immunoreactivity. (E, F) Higher magnification regions demonstrating low Ki‐67^+^ proliferation index throughout and monolayered rosettes that are negative for Ki‐67 (orange arrows) and one that shows Ki‐67 positive immunoreactivity (blue arrows).

Following the first resection of tumor tissue, and prior to chemotherapy, pathological examination of the collected specimen revealed a plethora of characteristic multilayered rosettes in a background of primitive, undifferentiated cells with high mitotic activity, atypia, and overabundant cellular density (Figure 3A–C). The tumor tissue was overcrowded with small blue embryonal cells with scant cytoplasm in the setting of hemorrhage, extensive angiogenesis, and an absence of mature cells (Figure 3A–C). Following four cycles of chemotherapy and the second surgical resection, the patient's pathology had noticeably changed. The residual tumor tissue was hypocellular, had fewer areas of angiogenesis; the cells were more differentiated and organized in a background of neuropil (Figure 3D–F). Furthermore, there were no multilayered rosettes observed; instead, the rosettes were monolayered, representing mature ependymal rosettes (Figure 3E,F). Differentiated neuronal cells were observed throughout the tumor tissue (Figure 3F). The Ki‐67 proliferation index was also higher at 60%–80% in the pre‐chemotherapy specimen (Figure 4A), with positive immunoreactivity detected in the cells of the multilayered rosettes and in the embryonal cells throughout the tissue (Figure 4B,C). This is in comparison to the post‐chemotherapy specimen, where the Ki‐67 proliferation index was <10%, and the monolayered rosettes were predominantly negative for Ki‐67 staining, with the exception of a few scattered positive monolayered rosette structures (Figure 4D–F).

### 2.4 Maintenance and patient follow‐up

Final treatment included high‐dose chemotherapy and stem cell rescue (HDCT/SCR) with a carboplatin and thiotepa conditioning regimen, with maintenance chemotherapy for 18 months with DFMO, everolimus, and intrathecal topotecan. The patient is now 6 years old (5 ½ years from diagnosis) and attends school. The patient requires hearing aids secondary to cisplatin‐related ototoxicity, which further highlights the need for less cytotoxic therapies in the pediatric population.

## 3 DISCUSSION

The case presented here is the fourth known case in the literature describing a patient with ETMR, whose post‐treatment pathology showed differentiation of tumor cells along their programmed lineage; this is in comparison to the pre‐treatment pathology of rapidly dividing undifferentiated cells. Importantly, to the best of our knowledge, this is the only report of an ETMR patient with post‐treatment differentiation of tumor cells who has exceeded the 5‐year survival mark following initial diagnosis.

Of the three post‐treatment differentiation cases published to date for ETMR, the study by Antonelli et al.^11^ is the only other case reporting long‐term patient survival. At the time of their publication, the patient was alive and well 31 months from initial diagnosis.^11^ The authors presented a case of a 21‐month‐old male diagnosed with ETMR predominantly in the left fronto‐opercular region.^11^ Histopathology post‐chemotherapy revealed the presence of neurons and ganglion cells within abundant neuropil, similar to the pathology findings reported here.^11^ Multilayered rosettes and immature embryonal cells were not detected, and amplification of the C19MC locus was only detected in about 10% of tumor nuclei, compared to 60% in the pre‐treatment tumor specimen.^11^ Interestingly, post‐treatment MRI of the case presented herein and the case presented by Antonelli et al.^11^ both demonstrated a significant reduction in tumor following therapy. We agree with the findings by Antonelli et al.^11^ in that the reduction in tumor burden is associated with neuronal maturation of the tumor compartment and loss of embryonal components seen in pre‐treatment tumor tissue, all of which are potentially associated with the improved clinical outcome observed in both cases.

Moreover, our case contributes to the growing number of reports of post‐treatment differentiation in embryonal tumors in general. In addition to this case, Levine et al.^12^ described the case of a 3‐year‐old girl with a pontine brain tumor originally thought to be a diffuse midline glioma versus a central primitive neuroectodermal tumor (PNET), but confirmed postmortem to be ETMR. While the pontine mass was slightly reduced in size after 6 months from the initial diagnosis and treatment, there was widespread dissemination elsewhere, and the patient eventually succumbed to her disease course.^12^ Upon postmortem brain biopsy, there were regions of primitive, proliferating, undifferentiated cell populations, but also less cellular regions with glial and neuronal maturation in the setting of a rich neuropil landscape.^12^ Only the primitive regions had strong immunoreactivity for LIN28A and only rare GFAP/synaptophysin positivity; the differentiated regions had strong immunoreactivity with MAP2 and GFAP, which are mature neuronal and glial markers, respectively, and these areas were completely negative for LIN28A.^12^

Furthermore, Lafay‐Cousin et al.^13^ described a 21‐month‐old female with a large, left frontoparietal tumor who underwent emergency gross total resection in the setting of uncal herniation. The pathology showed diffusely‐positive LIN28 immunoreactivity in the background of poorly differentiated neuroepithelial cells with rosettes that were not multilayered, along with minimal neuropil.^13^ FISH analysis of the 19q13.41 region was positive, consistent with ETMR despite the absence of multilayered rosettes.^13^ Following chemotherapy, imaging was suspicious for recurrence, with recurrent tumor discovered on second‐look surgery, showing features of maturation and hypocellularity in the background of abundant neuropil without areas of small, round, blue cells or rosettes, resembling a low‐grade glioneural tumor.^13^ Ganglion cell morphology and a low Ki‐67 index were both present, as was also seen in the case that we report here.^13^ Additionally, LIN28 was predominantly negative in these tumor samples.^13^ Following the second recurrence, however, the pathology resembled the pre‐treatment pathology, with undifferentiated tissue, rosettes, and high LIN28 positive immunoreactivity.^13^ This patient eventually died from her disease course.^13^

Interestingly, both Levine et al. and Lafay‐Cousin et al. present cases of patients who did not have impressive post‐treatment reduction in tumor bulk, both of whom had recurrence that eventually led to patient demise. Conversely, in the case that we present and in Antonelli et al., there were more impressive reductions in tumor bulk following chemotherapy treatment, and the patients had longer‐term survival. However, in all four cases, it is apparent that the molecular footprint of ETMR destines it for neuronal maturation, and the maintenance and extent of that maturation may dictate stability and survival.

Other instances of pediatric embryonal tumors undergoing post‐treatment maturation include: medulloblastoma, neuroblastoma, and high‐grade neoplasm with PNET‐like features. Wu et al.^14^ reported 2 patients with medulloblastoma with extensive nodularity, whose post‐chemoradiation pathology showed tumor cell maturation into a gangliocytoma. These patients had impressive reduction in tumor size from pre‐ to post‐treatment, and the patients were alive and well 6 and 3 years, respectively, at last follow‐up at the time of this publication.^14^ The authors also reported six other cases in the literature of patients with medulloblastoma who had post‐treatment maturation of tumor specimens into neuronal/glial, neuronal, gangliocytoma, ganglioneurocytoma, and ganglioglioma phenotypes across studies.^14^

Additionally, Bidgoli et al.^15^ reported on a 7‐week‐old female diagnosed with an embryonal tumor defined as PNET/cerebral neuroblastoma. Post‐chemotherapy, the resected tumor tissue was no longer WHO Grade IV, but a WHO Grade I ganglioglioma, inferring maturation and differentiation from the original tumor tissue biopsied pre‐treatment.^15^ As with all of the cases discussed thus far, there was impressive regression of tumor bulk. Additionally, the patient was alive and well over 3 years from initial diagnosis at the time of publication.^15^ Driever et al.^16^ reported a case of a 5‐year‐old male who was diagnosed on histopathology with an undifferentiated WHO Grade IV PNET with focal neuronal differentiation. Following chemoradiation, a local recurrence of the tumor showed loss of the initial small, round, blue cells and expression of glial fibrillary acid protein (GFAP), inferring differentiation along the astrocytic lineage.^16^ At the time of this publication, the patient was in complete remission 31 months from initial diagnosis.^16^ Lastly, Nozza et al.^17^ reported an 8‐month‐old male diagnosed with the malignant embryonal tumor, pineoblastoma, which histopathologically appears as small, round, blue cells akin to a PNET's histopathology. The histopathology seen on pre‐treatment biopsy specimens was consistent with a pineoblastoma; however, following treatment with inductive and high‐dose chemotherapy, repeat biopsy specimens revealed a neuronal tumor composed of ganglion and neurocytic cells, classified as a low‐grade neuronal tumor.^17^ The patient was still in remission 9 months following the end of therapy at the time of publication.^17^

Our case contributes to the relatively few pieces of literature that elucidate the clinical association of post‐treatment differentiation of tumor tissue on pathology, reduced tumor burden on imaging, and prolonged survival of the patient. A pre‐clinical correlate can be seen with the findings of Jessa et al.,^6^ who found a common theme of stunted differentiation of neural progenitors across various pediatric brain tumors; when a knockout mutant tumor line was allowed to differentiate, there was a phenotypic switch towards mature and differentiated cells. Given such findings, these investigators suggest that future therapeutic development be aimed at the differentiation of tumor tissue.^6^ Recently, brain‐penetrating imipridones (ONC201 and ONC206) were studied in in vitro and in vivo models of diffuse midline glioma (DMG), and Przystal et al.^18^ found that in a population of DMG cells that were treated with ONC201, there was a shift from an immature, undifferentiated oligodendrocyte‐like progenitor cell phenotype to an increased population of differentiated cells along the astrocytic lineage. Importantly, the findings of Przystal et al.^18^ led to the development of two clinical trials covering a number of different pediatric brain tumors.

In summary, we have presented a case with prolonged survival (>5 years) after post‐treatment differentiation of the patient's pathology and reviewed similar accounts reported in the literature. These collective findings support the notion for the development of novel therapeutics that are less cytotoxic than chemotherapy, but that achieve the objective of inducing maturation of embryonal tumor tissue in order to potentiate cancer remission.

## AUTHOR CONTRIBUTIONS

Francesca M. Gualano contributed to conceptualization, validation, writing (original draft, review, and editing), and visualization. Patrice Hassoun contributed to visualization and review. Claire L. Carter contributed to conceptualization, validation, writing (review and editing), visualization, supervision, and project administration. Derek Hanson contributed to conceptualization, validation, writing (review and editing), visualization, supervision, and project administration.

## CONFLICT OF INTEREST STATEMENT

The authors have stated explicitly that there are no conflicts of interest in connection with this article.

## ETHICS STATEMENT

We obtained a written statement of informed consent from the patient's parents for the publication of case details and use of images. The case discussed in this manuscript does not include patient‐identifying information, nor does it report a new study that required IRB approval.

## Data Availability

Data sharing is not applicable to this manuscript, as no new datasets were generated or analyzed during the current study.
